# Intoxication by Self-administered Cesium Salts, the Clinical Impact of Questionable Research Output

**DOI:** 10.1007/s12012-025-10081-9

**Published:** 2026-01-05

**Authors:** Jasmijn Brouwer, Soumaya Asaggau, Marjan Wafa, Julia P. J. Janssen, Willem Y. Hament, Hiela Bazgarjar, Kim G. Zwinkels, Ruben A. van Diest, Marcel A. G. van der Heyden

**Affiliations:** 1 Honours Program CRU+ Bachelor, University Medical Center Utrecht, Utrecht, The Netherlands; 2 Department of Medical Physiology, Division of Heart & Lungs, University Medical Center Utrecht, Yalelaan 50, 3584 CM Utrecht, The Netherlands

**Keywords:** Cesium, Ion channel, Poisoning, Intoxication, Misinformation

## Abstract

Cesium chloride (CsCl) is a non-radioactive salt wrongly promoted and used as part of an alternative cancer treatment based on questionable research output published in the mid-eighties. Self-administered cesium can lead to various symptoms. We analyzed the complete set of published case reports of people who have taken cesium to characterize demographics, reasons for intake, clinical effects, reported symptoms, pathophysiology, treatment options, and outcomes, followed by a historical and critical note. A total of 20 cases were included in this literature review. Most patients were females (n = 14), and almost half of the patients were between 40 and 49 years. Most patients used cesium as an alternative treatment for cancer (n = 15). When the route of administration was mentioned, it was most often oral, followed by intravenous use and combined routes. Symptoms occurred across multiple organ systems, including the cardiovascular, neuromuscular and gastrointestinal system. When ECG results were presented, QT prolongation, followed by sinus bradycardia and Torsade de Pointes arrhythmias were most often described. A wide variety of treatments have been provided to the patients. Five patients were reported to have died because of the cesium intake. After absorption, cesium is distributed throughout the body, where it inhibits ion channels, mainly for potassium. These channels, particularly in cardiac cells, are crucial for maintaining normal electrophysiology. The improper promotion of self-administration of cesium as part of an alternative cancer treatment, based on uncorrected scientific misinformation may result in life-threatening cardiac arrhythmia.

## Introduction

Cesium is an alkali metal that belongs to the same group as lithium, sodium, potassium, and rubidium. Due to the chemical and physical similarities among these elements, cesiums interaction with the human body, particularly in the context of its accumulation and excretion, is closely related to potassium. Cesium mimics potassium ions and will disrupt normal cellular processes, particularly those involving potassium ion channels. This can have significant clinical consequences, especially within the cardiovascular system [1].

Cesium chloride (CsCl) has been promoted as an alternative cancer treatment since the 1980s [2, 3], coined as “High pH Therapy” by Aubrey K. Brewer in 1984 [2]. CsCl was suggested to elevate the intracellular pH of tumor cells and eliminate toxins generated by tumor cells, thereby inhibiting their ability to divide and thus shorten their lifespan. Importantly, no solid clinical research has confirmed these claims. Remarkably, at about the same time, several research groups worked on the development of a canine cardiac arrhythmia model based on CsCl injection, using similar doses as promoted in “High pH Therapy” [4–7], which at time already should have raised questions about the safety and potential side effects of the promoted therapeutic CsCl application.

Since then, many cancer patients who do not wish to have the standard treatment, or have not found success with it, may turn to alternatives like CsCl. Information from various internet sources, private clinics, and books, promote such unproven therapies, including its dosing, for example [8]. CsCl remains readily available for purchase online via platforms such as Amazon, AliExpress, Etsy, and eBay, with prices ranging from €0.76 to €19.59 per gram when searched for on November 14th, 2024, and often without any warning of its pro-arrhythmic effects, e.g. [9]. This widespread availability enables self-administration by patients, often without medical supervision or awareness of the associated risks. From 1984 onwards, a number of case reports have been published on cesium intoxication and its frequent lethal outcome.

This study aims to analyze the complete set of published case reports, in any language, of people who have taken cesium. It will explore the reasons for intake, clinical effects, reported symptoms, pathophysiology, and treatment options. This will provide a more comprehensive understanding of the health risks related to cesium intake, offer valuable insights for healthcare providers, and depicts the serious consequences of scientific misinformation.

## Methods

We conducted a literature search of case reports on cesium chloride or carbonate intake using PubMed and Google Scholar, including articles published on these platforms up to September 16, 2024. The search terms used in PubMed included: ‘Cesium chloride "case report"’, ‘CsCl case report’, ‘Alternative cancer therapy cesium case report’, ‘Cesium chloride hypokalemia’, ‘Cesium carbonate "case report"’, and ‘Cesium carbonate hypokalemia’. The search terms used in Google Scholar were: ‘“cesium chloride” torsade de pointes “case report”’, ‘"cesium intake" "case report"’, ‘"CsCl" "case report" arrhythmia cesium’, and ‘"CsCl" "cesium chloride" "case report" ventricular arrhythmia’.

**Fig. 1:**
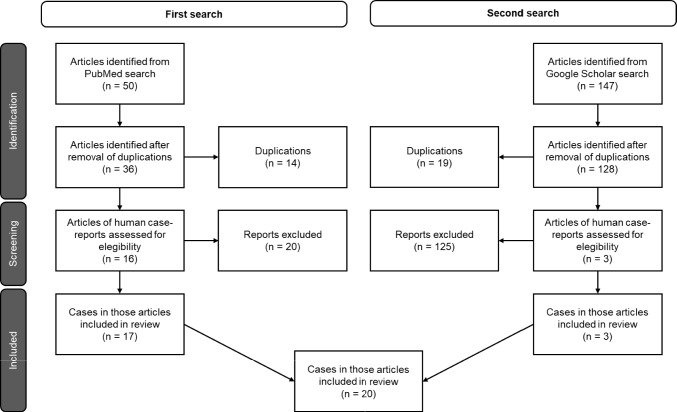
Flowchart depicting the search and selection process for case reports of patients who took cesium. The initial PubMed search yielded 50 results, reduced to 16 case reports, describing 17 cases. A second search in Google Scholar identified 147 articles, which after removing duplications and non-case reports yielded 3 not previously identified articles. In total, 20 cases were included in this literature review

Additional case reports were identified by reviewing the reference lists of the primary articles retrieved. Only articles containing full-text case reports on patients taking non-radioactive cesium were included in the analysis. Exclusion criteria included articles on patients who had been exposed to radioactive cesium, studies involving animals, and duplicate publications or those reporting the same cases. A total of 20 cases were included in this literature review (Fig. 1). All included reports were written in English, except for one Norwegian article, which was reviewed in its original language by the current authors and partially translated using Google Translate.

From each case, we collected data on patient demographics (age, sex), medical background, cesium dosage, route and frequency of administration, maximum corrected QT interval (QTc), electrocardiogram (ECG) findings, blood values, clinical symptoms, treatments, duration of hospitalization, and the outcome of the case. We also documented the geographical origin of the article, determined by the location of the first author.

Each of the collected case reports was independently reviewed by at least two authors, who reached a consensus regarding the interpretation of the data.

## Results

**Fig. 2:**
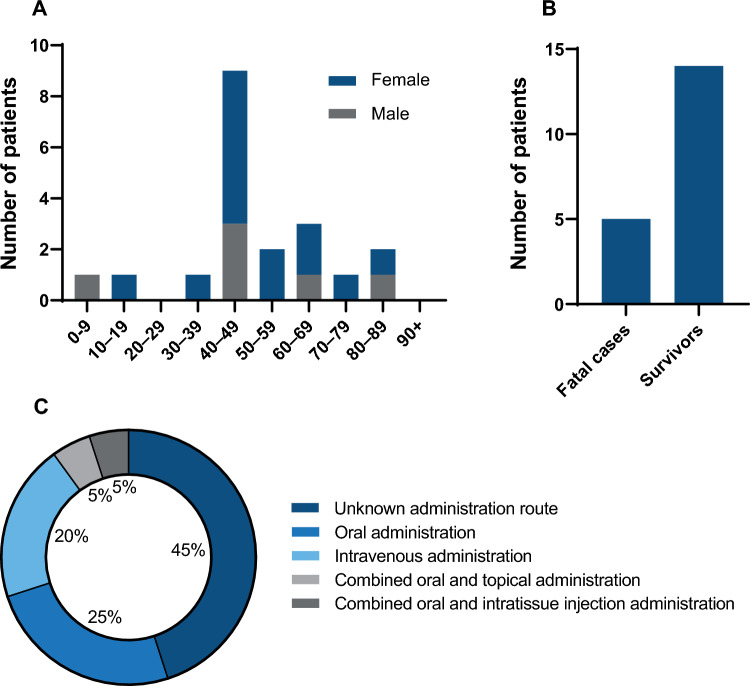
**A** Age and sex distribution of cesium intoxication cases. **B** distribution of clinical outcome. **C** Routes of cesium salt self-administration

The search for cesium salt intoxication cases yielded 19 reports presenting a total of 20 individual cases (Fig. 1, Table 1) [10–28]. The vast majority of these cases originated from North America, predominantly from the United States of America and Canada (Table 1). Most patients were female, with nearly half aged between 40 and 49 years (Fig. 2A). Cesium intoxication resulted in fatal outcomes in five patients (Fig. 2B). Of the five fatal cases, two resulted from cardiac arrest, one from an unspecified cardiac arrhythmia, and one occurred during hospitalization after a seizure, during which the patient developed sepsis and multiple subsequent seizures. In the remaining fatal case, the cause of death was not documented.

**Table 1:** Overview of reported cases involving cesium use

Case no.	Patient charact	Reason for intake	Outcome	Mean dose(g/Day)*	Origin of publication	First author	Year of publication
Case 1	41/M	Self-experiment	Survived	6	USA	Neulieb [10]	1984
Case 2	47/F	Prevention: Breast Cancer	Survived	3	USA	Saliba [11]	2001
Case 3	62/M	Treatment: Prostate Cancer	Survived	4	Canada	Pinter [12]	2002
Case 4	52/F	Treatment: Colon Cancer	Survived	3	Canada	Lyon [13]	2003
Case 5	41/M	Treatment: Kidney Cancer	Died	Not Reported	USA	Centeno [14]	2003
Case 6	82/M	Treatment: Lung Cancer	Died	Not Reported	USA	Centeno [14]	2003
Case 7	43/F	Treatment: Glioblastoma	Survived	9	USA	Dalal [15]	2004
Case 8	8/M	Treatment: Osteogenic Sarcoma	Survived	Not Reported	USA	Curry [16]	2006
Case 9	39/F	Menorrhagia	Survived	Not Reported	USA	Vyas [17]	2006
Case 10	55/F	Treatment: Ovarian Cancer	Died	7.5	USA	Nuwayhid [18]	2007
Case 11	16/F	Treatment: Liver Cancer	Survived	3	USA	O’Brien [19]	2008
Case 12	84/F	Treatment: Kidney Cancer	Not Reported	3	Canada	Painter [20]	2008
Case 13	65/F	Treatment: Rectal Cancer	Survived	Not Reported	Hong Kong	Chan [21]	2009
Case 14	45/F	Treatment: Breast Cancer	Survived	3	Canada	Wiens [22]	2009
Case 15	46/F	Treatment: Melanoma	Survived	10	Canada	Young [23]	2013
Case 16	61/F	Treatment: Breast mass	Died	Not Reported	USA	Sessions [24]	2013
Case 17	42/F	Treatment: Breast Cancer	Died	Not Reported	Australia	Khangure [25]	2013
Case 18	40/F	Treatment: Rectal Cancer	Survived	9	Norway	Warsame [26]	2014
Case 19	45/M	Treatment: Laryngeal Cancer	Survived	3	USA	Horn [27]	2015
Case 20	77/F	Treatment: Colon Cancer	Survived	0.5	USA	Hetavi [28]	2019

^*^In cases 15 and 18 cesium-carbonate was used, in all other cases cesium-chloride was taken

The primary indication to take cesium was cancer-related, reported in 18 cases: 16 patients used cesium as an alternative cancer treatment, one for cancer prevention, and one for treating a tumor of unknown malignancy (Table 1). In two isolated cases, cesium was taken either as part of a self-experiment or to manage menorrhagia.

When reported, the route of cesium administration was predominantly oral or intravenous (Fig. 2C). Occasionally, oral administration was combined with topical application or intratissue injection. Among the five fatal cases, cesium was administered intravenously in three, a combination of oral and intratissue injection was used in one, and in the remaining case, the route of administration was not documented. Cesium was most commonly administered in the form of cesium chloride (CsCl) (n = 16). In two cases, cesium carbonate (Cs_2_CO_3_) was used, while in another two cases the specific chemical form of cesium was not documented. The ingested dose of cesium, administered either as CsCl or Cs_2_CO_3_, was documented in eleven cases (Table 1). The median daily dose was 23.8 mmol Cs⁺ (interquartile range [IQR], 17.8 – 53.5 mmol/day) (Table 2). In four of the five fatal cases, the dose was not documented.

**Table 2:** Serum potassium, QTc interval duration and internalized dose of cesium-ions

Case no.	Serum potassium (mmol/L)	Maximum QTc interval (ms)	Cs^+^ Mean dose (mmol/day)
Case 1	–	–	36.8
Case 2	3.2*	691	17.8
Case 3	2.8	–	23.8
Case 4	2.8	580	–
Case 5	–	–	–
Case 6	–	–	–
Case 7	3.1	624	53.5
Case 8	–	710	–
Case 9	3.1	616	–
Case 10	–	–	44.6
Case 11	–	683	17.8
Case 12	–	–	17.8
Case 13	2.8	620	-
Case 14	3	516	17.8
Case 15	3.7	620	61.4**
Case 16	2.7	694	–
Case 17	–	–	–
Case 18	2.8	596	55.2**
Case 19	3.3	–	17.8
Case 20	3.5	–	–

^*^Potentially misreported as mg/dL by the authors

^**^Cesium was administered as cesium carbonate in cases 15 and 18; in all other cases, cesium chloride was used

Hypokalemia was observed in 10 of 12 patients with reported serum potassium levels, with a median concentration of 3.05 mmol/L (IQR, 2.8 – 3.25 mmol/L) (Table 2). A serum potassium level was documented in only one of the five fatal cases, measuring 2.7 mmol/L.

**Fig. 3:**
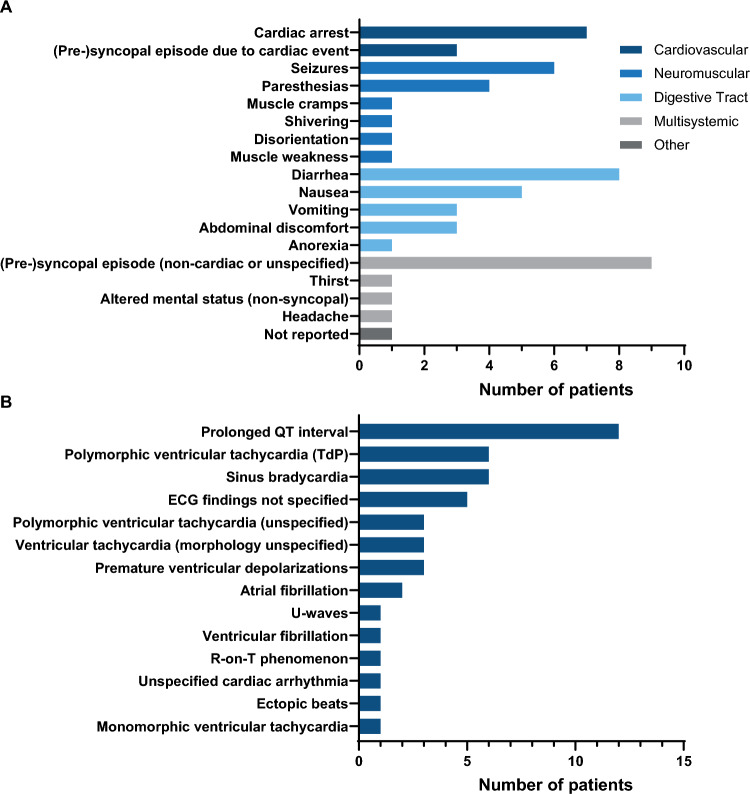
**A** Frequency of clinical symptoms following cesium intoxication. **B** Frequency of observed ECG characteristics. In five cases, no ECG data are reported

Clinical manifestations following cesium intake affected multiple organ systems, including cardiovascular, neuromuscular, and gastrointestinal systems (Fig. 3A). Cardiac arrest occurred in seven cases, while presyncopal or syncopal episodes attributable to cardiac events were reported in an additional three cases. Another 9 patients experienced presyncopal or syncopal episodes without a specified underlying cause. Seizures were documented in 6 patients. Less severe but frequently reported symptoms included diarrhea, nausea, and paresthesias. In one case, no symptoms were reported.

In association with half of the cases presenting with cardiovascular effects of cesium, 13 different ECG changes were reported in 15 patients (Fig. 3B). The most frequently observed abnormality was QT-interval prolongation, described in 14 cases. QTc interval durations were provided in eleven of these cases, with a median QTc of 620 ms (IQR, 596–691 ms) (Table 2). Polymorphic ventricular tachycardia was reported in nine cases, with Torsades de Pointes (TdP) specifically identified in six. In the remaining three cases, the polymorphic ventricular tachycardia subtype was unspecified. Monomorphic ventricular tachycardia was documented in one case, and in three cases ventricular tachycardia was noted without morphological details. Other ECG abnormalities included sinus bradycardia and premature ventricular depolarizations.

Treatment was documented in sixteen patients, addressing various clinical needs such as management of cesium exposure, electrolyte correction, cardiovascular and respiratory support, and seizure prophylaxis and treatment (Table 3). Cesium cessation was reported in eight cases to limit exposure, and in three cases, Prussian Blue was administered to facilitate cesium removal via ion exchange [29]. Potassium supplementation, often combined with magnesium supplementation, was given to correct hypokalemia. Cardiovascular support commonly involved lidocaine, cardiopulmonary resuscitation (CPR), and electrical cardioversion. Seizure prophylaxis was documented five times, all administered to a single patient.

**Table 3:** Frequencies of treatment modalities used

Treatment	Number of pts. (%)
Management of cesium exposure	
Prussian Blue	3 (15)
Cessation of cesium therapy	8 (40)
Electrolyte correction	
Na^+^ suppletion	3 (15)
K^+^ suppletion	12 (60)
Mg^2+^ suppletion*	9 (45)
Cardiovascular support	
CPR	5 (25)
Electrical cardioversion	4 (20)
Pacemaker	1 (5)
Amiloride	1 (5)
Amiodarone	3 (15)
Isoproterenol	2 (10)
Atenolol	1 (5)
Metoprolol	1 (5)
Adenosine	1 (5)
Lidocaine	6 (30)
Respiratory support	
Intubation	2 (10)
Anti-seizure	
Mannitol**	1 (5)
Dexamethasone**	1 (5)
Valproic acid	1 (5)
Levetiracetam	1 (5)
Lorazepam	1 (5)
Supportive care	
Cooling	1 (5)
Analgesics	1 (5)
Other	
No treatment reported	4 (20)

^*^In eight out of nine patients, magnesium was given in combination with potassium suppletion

^**^Given because of a high intracranial pressure

*CPR* Cardiopulmonary resuscitation

## Discussion

We reviewed case reports of non-radioactive cesium poisoning resulting from self-administration. The majority of patients, we are inclined to call them victims, took several grams of CsCl in an attempt to treat their diagnosed cancer, as promoted on various websites and books, without supervision of a certified clinician. Many patients experienced cardiac arrhythmia, (pre)syncope and five (25%) died due to the cesium intake, as stated in the case reports.

When ingested, cesium is almost completely absorbed from the small intestine [1]. Cesium is mainly excreted via the kidneys and has a half-life of approximately 2 to 4 months [30, 31]. This long half-life may prolong the occurrence of toxic effects or adverse reactions. Following absorption, cesium is distributed throughout the body, where it inhibits ion channels, mainly for potassium. This potassium ion channel blockade most likely stands at the basis of many of the reported symptoms following cesium intoxication.

Inhibition of the delayed rectifier potassium channels in the myocardium results in QT prolongation on the ECG, reported in 60% of the case reports. QT prolongation is an established risk factor of ventricular cardiac arrhythmias [32]. In preclinical research that used six healthy dogs (10–20 kg), a bolus injection of 1.7 to 3.4 g (1 mmol/kg) resulted in a transient QT prolongation, and following a second bolus after 10 min, all animals developed ventricular arrhythmia [6].

Potassium inward rectifier channels of the *KCNJ* gene family are also targeted by cesium resulting in channel inhibition in the micromolar range [33]. This diverse inward rectifier potassium channel family has roles in multiple organ systems (neural, kidney, heart) and its inhibition results in, for example, unstable plasma-membrane potential in neural and cardiac cells, and disturbances in renal electrolyte handling [34, 35]. The latter likely involves inhibition of the inward rectifier channels Kir1.1 and Kir4.1, resulting in excessive renal potassium wasting, inducing hypokalemia, which contributes to the QTc prolongation [36]. Furthermore, cesium may induce hypokalemia by inhibiting channels responsible for potassium uptake from dietary sources. Finally, potassium depletion may occur through increased gastrointestinal losses as associated with the frequently observed diarrhea (Fig. 3A). Two mechanisms have been described for the blockade of KCNJ channels. The first mechanism, called the "obstruction mechanism", occurs when cesium ions align with potassium ions within the selectivity filter of the Kir channel. Potassium ions can easily overcome energy barriers within the channel, allowing for the passage of potassium ions across the membrane. In contrast, cesium ions are unable to overcome all the necessary energy barriers, resulting in them being "trapped" within the channel. This trapping prevents potassium ions from moving across the membrane, thereby inhibiting potassium influx [37–39]. In the second mechanism, cesium ions likely bind to common sites on the Kir channel outside the selectivity filter, preventing the outward movement of potassium ions [40].

Finally, pacemaker channels of the HCN family become also inhibited by cesium in the low millimolar range [33, 41]. It cannot be excluded that the observed sinus bradycardia in six patients relates to this pacemaker channel inhibiting effect.

### Misconception of cesium as alternative cancer therapy, history and critical note

The use of cesium as an alternative cancer treatment originates from a misinterpretation of early hypotheses concerning cancer cell metabolism. In 1956, Dr. Otto Warburg, a pioneer in the field of oncometabolism and Nobel laureate, observed that cancer cells rely heavily on anaerobic glycolysis for energy production; a phenomenon that results in elevated lactic acid levels. Warburg postulated that this metabolic shift played a causative role in malignant transformation [42]. However, research has since demonstrated that such alterations in metabolism are secondary to genetic mutations in oncogenes and tumor suppressor genes, rather than a primary cause of cancer pathogenesis [43, 44]. Nonetheless, misconception of the Warburg’s hypothesis still feeds false claims, for instance in popular books and social media, on the efficacy of dietary therapies and their dangerous consequences [45]. It also served as the foundation for so-called “High pH Therapy”, introduced by Dr. Aubrey Keith Brewer [2]. This alternative medicine therapy was first published in a supplement of *Pharmacology Biochemistry and Behavior*, edited by F.S. Messiha in 1984, which features several papers on CsCl-based “High pH Therapy” by invited experts and participants of the 2nd Annual Toxicology Conference (October 1983, El Paso, Tx, USA). Among them were empirical studies, theoretical discussions and the first case report on self-administered CsCl [10].

Brewer hypothesized that increasing intracellular pH through administration of CsCl could inhibit cancer progression [2]. In his publications, he described tumor regression in mice within two–three weeks of cesium/CsCl administration and reported complete tumor disappearance and symptom resolution within 12 to 24 h in 30 human cancer patients [2, 46]. This rationale was echoed by Sartori, who outlined the theoretical basis for cesium therapy in cancer [47]. Additionally, Sartori presented a case series involving 50 patients with advanced, metastatic disease [3]. He claimed a 50% recovery rate and consistent pain relief within 1 to 3 days of initiating CsCl therapy. The side effects Sartori and Brewer reported were limited to loss of appetite, nausea, diarrhea, and perioral paresthesia. Furthermore, they administered doses up to 30 g/day, significantly exceeding then-available protocol recommendations of 6–9 g/day. Sartori was later convicted of practicing medicine without a license, further undermining the credibility of his findings [48].

Neither Brewer’s nor Sartori’s results were presented with robust data, long-term follow-up or have ever been independently verified in other studies. While their publications are sometimes briefly mentioned in reviews or used as contextual background for investigations into other metals (e.g., rubidium, magnesium and potassium), multiple sources critically describe their work [1, 49, 50]. Nonetheless, their claims have remained influential and are frequently cited in support of cesium therapy for cancer. Of the 19 case-report articles reviewed for this study, 16 referenced “High pH Therapy” or directly cited Sartori’s and Brewer’s publications, often as the sole justification for using cesium therapeutically.

In the Netherlands, the Nationaal Vergiftigingen Informatie Centrum has reported no cases involving cesium intoxication [51]. Moreover, no data on the number of cesium users is available, but in view of additional cases briefly mentioned by the FDA [52], we may assume many more intoxications do occur. In light of mounting evidence of toxicity, most notably severe cardiac arrhythmias, the U.S. Food and Drug Administration (FDA) issued several public health warnings between 2018 and 2023 [52–55]. These warnings explicitly advise against the use of cesium for any therapeutic purpose and sale of CsCl-containing supplements. In 2023, the FDA reaffirmed that CsCl is a new dietary ingredient for which no adequate evidence of safety has been provided. As such, dietary supplements containing CsCl are considered adulterated under the Federal Food, Drug, and Cosmetic Act. Similarly, cesium is not listed in the European Union’s Directive 2002/46/EC, which defines approved substances for use in food supplements. While this does not constitute an outright ban, it renders its sale as a supplement unlawful under current federal regulations in the US and EU. Even more encouragingly, only one case report has been published since the issuance of the first FDA warning in 2018 [28].

This review exemplifies the far-reaching consequences of unchecked and non-corrected scientific misinformation. Despite earlier publications on the consequences of CsCl administrations as that of Melnikov in 2010 [1], the continued citation and application of flawed studies not only erode the integrity of scientific discourse, but also pose direct threats to patient safety, as becomes evident from the publication of six case reports since, of which two reporting a lethal outcome [23–28]. Although correcting scientific knowledge over time is part of the scientific process, studies indicate that publications remain being cited in scholarly publications after their retraction [56]. Moreover, non-scholarly publications still refer to “High pH Therapy” work as “scientifically proven” as long as the work exists in its current form.

Here, we highlight several methodological weaknesses, 20 case reports and repeated health warnings for a supplement of the journal *Pharmacology Biochemistry and Behavior*. We therefore plea for the release of an editorial note or expression of concern on the entire issue, in line with the COPE Guidelines on Expressions of Concern, Position on old papers [57, 58]. Similar foundational studies on alternative medicine therapies have been retracted previously [59, 60].

## Conclusion

Self-administration of cesium salts is often done for alternative treatment of cancers of various types, promoted in books and social media as high-pH therapy, but without any solid clinical research. Most case reports originate from North America and show an overrepresentation of the 40–49-year age group. Many patients develop serious neurological and cardiac symptoms, like seizures, syncope, ventricular arrhythmia and cardiac arrest. Electrolyte correction, cardiovascular support and cessation of cesium therapy are the mostly provided treatments. Twenty-five percent of the presented cases had a fatal outcome. The continued presence of non-corrected scientific misinformation in the life sciences scholarly literature can lead to serious health issues.

## Data Availability

Data will be made available on request.
